# Comparative Analysis of Antimicrobial Resistance Profiles in Enterobacteriaceae Isolates From Outpatient Carriages and Clinical Infections in an Urban Setting

**DOI:** 10.1155/bmri/8393896

**Published:** 2026-08-12

**Authors:** Kobina Amandzé Adams Kofi, Stéphane Kouadio Koffi, Mathieu Kra Adou Koffi, Alexia Yapi Ivan, N′guessan Carole Obouayeba Djaman, Aboubacar Bamba, Flora Ahonzo, Kacou N′douba Adèle

**Affiliations:** ^1^ Biology and Health Laboratory, Biosciences Training and Research Unit, Université Félix Houphouët-Boigny, Abidjan, Côte d’Ivoire, univ-fhb.edu.ci; ^2^ Bacteriology-Virology Unit, University Hospital of Treichville, Abidjan, Côte d’Ivoire; ^3^ Medical Sciences Training and Research Unit, Université Félix Houphouët-Boigny, Abidjan, Côte d’Ivoire, univ-fhb.edu.ci

**Keywords:** antimicrobial resistance dynamics, clinical infections, community–hospital surveillance, intestinal carriage strains, multidrug-resistant Enterobacteriaceae

## Abstract

**Background:**

The emergence and spread of multidrug‐resistant Enterobacteriaceae (MDR‐E) in community and hospital settings constitute a major public health challenge, particularly in densely populated urban areas. Although antimicrobial resistance among clinical isolates has been repeatedly studied, resistance profiles of intestinal carriage strains remain scarcely reported, despite their potential role as important reservoirs of resistance genes. An integrated approach including both carriage and clinical isolates is therefore necessary to better understand antimicrobial resistance dynamics.

**Objective:**

This study is aimed at comparing the antibiotic resistance profiles of Enterobacteriaceae isolated from intestinal carriage in outpatients and those from clinical infections.

**Methods:**

A total of 196 strains, including *Escherichia coli*, *Klebsiella pneumoniae*, and *Enterobacter cloacae*, were analyzed, comprising 60 carriage isolates and 136 clinical isolates. Antimicrobial susceptibility testing was performed using the disk diffusion method according to CA‐SFM guidelines. Resistance profiles were classified as multidrug‐resistant (MDR), extensively drug‐resistant (XDR), or pandrug‐resistant (PDR) based on the criteria of Magiorakos et al., and the multiple antibiotic resistance (MAR) index was calculated.

**Results:**

Overall, a high prevalence of MDR strains was observed (86.73%) in both groups, with distinct resistance patterns depending on the source. Carriage strains showed higher resistance to cephalosporins, fluoroquinolones, monobactams, tetracyclines, and sulfonamides (MAR index: 0.76 ± 0.09), whereas clinical strains exhibited increased resistance to aminoglycosides and carbapenems (MAR index: 0.65 ± 0.18). XDR (6.12%) and PDR (1.02%) phenotypes were mainly identified among clinical isolates.

**Conclusion:**

These findings emphasize the role of intestinal carriage as a reservoir of antimicrobial resistance and support the need for integrated community–hospital surveillance strategies in urban settings.

## 1. Introduction

The spread of antimicrobial resistance is a major global health challenge. In 2022, approximately 1.27 million deaths were attributable to antimicrobial resistance, with an expected death rate of 27.3 per 100,000 people in sub‐Saharan Africa, the highest among all regions worldwide [1]. In response to this growing threat, various strategies have been implemented worldwide [2, 3]. Among these strategies, the surveillance of resistant strains is central, enabling the monitoring of the evolution of resistant strains in the community and hospitals, as well as guiding therapeutic and prevention policies.

Among the bacteria of particular concern, extended‐spectrum beta‐lactamase (ESBL)–producing Enterobacteriaceae represent a major public health problem because of their ability to hydrolyze penicillin, cephalosporins, and monobactams [4, 5]. This resistance is most often associated with genes located on large plasmids, which also carry resistance determinants to other classes of antibiotics, such as aminoglycosides, fluoroquinolones, tetracyclines (TETs), and sulfonamides, thereby considerably reducing treatment options [6, 7].

First identified in hospitals in the 1990s, ESBL‐producing Enterobacteriaceae have progressively spread into the community, demonstrating the transfer of resistance mechanisms from hospital settings [8, 9]. This community spread now poses a major challenge for the treatment of infections, as it leads to the silent circulation of resistance genes within the population and contributes to an increase in infections that are difficult to treat.

The spread of ESBL‐producing Enterobacteriaceae has been described in large‐scale studies at both hospital and community levels internationally [9, 10]. In Europe, although the prevalence of ESBL‐producing Enterobacteriaceae is low, an increasing prevalence has been reported among hospitalized patients and outpatients in the community, revealing a gradual spread of the problem beyond healthcare settings [11]. In Asia, a high prevalence has been reported, particularly in India and China, where more than 60% of Enterobacteriaceae strains isolated from the community carry ESBL genes [12]. In sub‐Saharan Africa, published data show a high prevalence in both hospitalized patients and the community, suggesting the silent circulation of these resistance genes in the community [10, 13].

In Côte d′Ivoire, many studies have documented the presence of ESBL‐producing Enterobacteriaceae in clinical infections, highlighting the rates of resistance in hospitals, which is a cause of considerable concern [14–16]. These infections are thought to represent part of a vast problem in which digestive tract carriage could be a key factor. In fact, the gut reservoir contributes to the silent spread and persistence of multidrug‐resistant (MDR) strains in both hospital and community settings [9].

The alarming progression of MDR bacteria is now a major public health issue, particularly in developing countries, where the lack of policies to control the use of antibiotics in humans and animals leads to strong selection pressure and promotes the emergence of resistant strains [17, 18]. In this context, the production of ESBL has become one of the most frequently observed resistance mechanisms [19, 20]. These enzymes are often associated with the presence of other resistance genes, resulting in MDR, extensively drug‐resistant (XDR), or pandrug‐resistant (PDR) profiles [21]. Furthermore, ESBL‐producing strains not only pose a direct therapeutic threat but also serve as indicators of the spread of bacterial antibiotic resistance in communities and hospitals [13].

However, despite this alarming context, available data in Côte d′Ivoire remain fragmented. Previous studies have mainly focused either on MDR Enterobacteriaceae isolated from clinical infections or on intestinal carriage among community populations; yet, none have investigated the potential contribution of carriage strains to the circulation of resistant bacteria implicated in clinical infections. To date, no study conducted in Côte d′Ivoire has directly compared resistant Enterobacteriaceae isolated from asymptomatic carriage with those isolated from clinical infections within the same epidemiological setting. A recent investigation published in 2023 explored community reservoirs of ESBL‐producing Enterobacteriaceae [22]; however, it was limited to carriage studies and did not assess the relationship between carriage strains and clinical isolates. This knowledge gap limits our understanding of transmission pathways and the role of asymptomatic carriage in the dissemination of MDR strains between community reservoirs and clinical settings.

Therefore, this study is aimed at comparing the resistance profiles of Enterobacteriaceae isolated from intestinal carriage in outpatients and those from clinical infections, providing further insights into the dynamics of antimicrobial resistance circulation between community and healthcare‐associated reservoirs.

## 2. Materials and Methods

### 2.1. Ethical Approval

This research was conducted in accordance with the Declaration of Helsinki and approved by the National Ethics Committee for Life Sciences and Health of Côte d′Ivoire (No. 223‐21/MSHPCMU/CNESVS‐Km) for the implementation of a research project on the circulation of MDR Enterobacteriaceae in humans, animals, and the environment in rural, peri‐urban, and urban areas of developing countries. Each study participant was informed of the objectives, collection methods, benefits, and risks of the study, and written informed consent was obtained from each study participant.

### 2.2. Study Design

**Figure 1:**
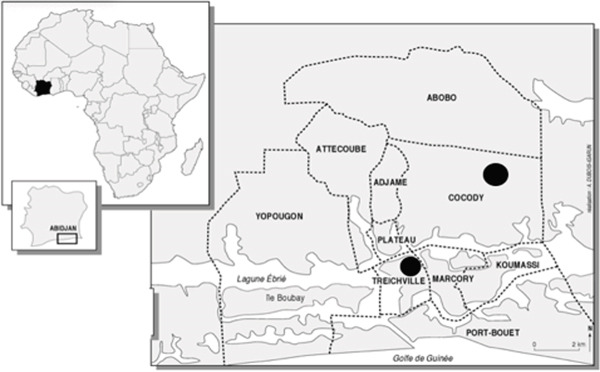
Study areas.

This was a cross‐sectional study with descriptive and analytical aspects, carried out on a total study period spanning from July 2022 to December 2023, in two healthcare centers in Abidjan: University Hospital of Treichville (5°17 ^′^28.28″ N, 4°0 ^′^12.83″ W) and University Hospital of Angré (5°24 ^′^3.65″ N, 3°57 ^′^26.77″ W) (Figure 1). Sample collection was performed exclusively during periods covered by valid ethical approval, and no sampling was conducted between January 2023 and November 2023. These centers were chosen for their high number of outpatients, high hospitalization rates, and technical capacity for microbiological analysis.

The centers were used to select participants and collect biological samples for the isolation of the strains studied in this work. Microbiological analyses were performed at the Bacteriology‐Virology Laboratory of the University Hospital of Treichville.

### 2.3. Sample Collection

Samples were collected from two groups of patients. The first group consisted of outpatients recruited from the external services of the two university hospitals for problems other than digestive disorders. Outpatients included in the study received standardized verbal instructions from trained healthcare personnel on how to perform self‐administered rectal swab sampling. Participants were instructed to wash their hands before sampling, open the package of sterile cotton‐tipped swabs with wooden shafts without contaminating the tip, gently insert the swab approximately 2 cm into the rectum, rotate it carefully three times to ensure adequate fecal material collection, and immediately place the swab into the sterile transport tube provided. Samples were returned immediately to the healthcare staff for transport to the laboratory.

Bacterial strains isolated from rectal swab in this group of patients were considered asymptomatic carriage strains.

The second group comprised hospitalized patients with bacterial infections. Bacterial strains isolated from clinical samples (such as blood, urine, and pus) obtained from this group were considered clinical strains.

### 2.4. Inclusion and Exclusion Criteria

For carriage strains, any *Escherichia coli*, *Enterobacter cloacae*, or *Klebsiella pneumoniae* were isolated from outpatients aged ≥ 15 years who had visited the consultation services of the sites in the study, had not taken any antibiotics in the 72 h prior to sampling, consented to participate in the study, and provided a stool sample via self‐administered rectal swab. Strains isolated from patients with diarrhea were not included in this study. The strains collected were stored in the laboratory at −20°C in heart–brain broth supplemented with 20% glycerol until analysis.

For clinical strains, any *E. coli*, *E. cloacae*, or *K. pneumoniae* isolated from hospitalized patients with confirmed infections and stored were included in this study. Duplicates considered to be the same strain isolated from the same patient during the same period were excluded from the study.

### 2.5. Microbiological Analysis

#### 2.5.1. Sample Processing and Culture

Immediately after collection, fecal samples obtained by rectal swabbing using sterile cotton‐tipped swabs with wooden shafts were placed into 2 mL of sterile physiological saline solution and transported to the laboratory within 2–4 h under refrigerated conditions (4°C–8°C).

Upon receipt in the laboratory, each sample was vortexed briefly to homogenize the fecal material in the saline solution. An aliquot of 10 *μ*L of this suspension was streaked onto Drigalski agar supplemented with ceftazidime (CAZ) (4 mg/mL) for selective isolation of resistant Enterobacteriaceae. The inoculated plates were incubated at 37°C for 18–24 h.

After incubation, colonies showing morphological characteristics compatible with Enterobacteriaceae were selected and subcultured onto nutrient agar plates to obtain pure isolates. Pure colonies were then subjected to Gram staining and conventional biochemical identification procedures.

For clinical specimens (blood, urine, and pus), routine microbiological processing was performed according to standard laboratory procedures.

#### 2.5.2. Strain Identification

Strain identification was performed using conventional bacteriological methods based on morphological characteristics (Gram‐negative bacilli, motile by peritrichous flagella or nonmotile), culture characteristics (smooth [S] or mucoid [M] colonies on eosin methylene blue agar), and biochemical characteristics using the Le Minor reduced biochemical identification scheme. The biochemical tests included glucose fermentation, gas production, hydrogen sulfide (H_2_S) production, urease activity, indole production, citrate utilization, lysine decarboxylase activity, ornithine decarboxylase activity, motility testing, lactose fermentation, mannitol fermentation, and oxidase testing.

#### 2.5.3. Antibiotic Susceptibility

Antibiotic susceptibility was determined using the Kirby–Bauer agar diffusion method, according to the 2024 recommendations of the Antibiogram Committee of the French Society of Microbiology (CA‐SFM) available at https://www.sfm-microbiologie.org/wp-content/uploads/2024/06/CASFM2024_V1.0.pdf. Antibiotic disks were placed on Mueller–Hinton agar plates inoculated with a 0.5 McFarland bacterial suspension. The antibiotics tested included amoxicillin (AMO 20 *μ*g), ticarcillin (TIC 75 *μ*g), amoxicillin–clavulanic acid (AMC 20/10 *μ*g), cefuroxime (CXM 30 *μ*g), cefoxitin (FOX 30 *μ*g), CAZ 10 *μ*g, cefotaxime (CTX 5 *μ*g), cefixime (FIX 5 *μ*g), cefepime (FEP 30 *μ*g), aztreonam (ATM 30 *μ*g), meropenem (MEM 10 *μ*g), and imipenem (IMP 10 *μ*g) for betalactamin. For quinolones, nalidixic acid (NAL 30 *μ*g) and ciprofloxacin (CIP 5 *μ*g) were tested, and gentamicin (GMN 10 *μ*g), TET 30 *μ*g, and cotrimoxazole (SXT 1.25/23.75 *μ*g) were tested for aminoglycosides, cyclins, and sulfonamides, respectively. After incubation at 37°C under aerobic conditions for 24 h, the diameters of inhibition were measured and interpreted according to the CA‐SFM guidelines, with a distinction between acquired and natural resistance, particularly for *E. cloacae* and *K. pneumoniae*.

To detect the presence of ESBL production, disks containing AMC and the cephalosporins ceftriaxone, CTX, CAZ, and FIX were placed on Mueller–Hinton agar at a distance of 20 mm edge‐to‐edge. Following incubation at 37°C for 24 h, the appearance of a synergy zone between any cephalosporin disk and the AMC disk was interpreted as positive for ESBL production. *K. pneumoniae* ATCC 700603 and *E. coli* ATCC 25922 were used as positive and negative control strains, respectively, for ESBL detection and antibiotic susceptibility testing.

#### 2.5.4. Classification of Multidrug Resistance Profiles

The resistance profiles of the strains were classified according to the consensus definition proposed by Magiorakos et al. [17], which allowed the identification of three levels of multidrug resistance. The strains were classified as MDR when they were resistant to at least one of three different classes of antibiotics. The strains considered XDR were those that were resistant to all classes of antibiotics tested, with the exception of one or two classes of antibiotics. PDR strains were defined as those resistant to all classes of antibiotics. This classification was applied to all Enterobacteriaceae strains included in the study, both from carriage and clinical infections.

#### 2.5.5. Determination of the Difference (*Δ*) in Resistance Levels and the Multiple Antibiotic Resistance (MAR) Index

For each class of antibiotic analyzed, the difference in resistance levels was determined using the following equation:

Δ %=T carriage strains−T clinical strains,

where *Δ* (*%*) represents the percentage difference and *T* represents the resistance level.

The MAR index was determined for each isolate via the following formula:

MAR=ab,

where *a* represents the number of antibiotics to which the strain was resistant and *b* represents the total number of antibiotics tested. An MAR index greater than 0.2 reflects exposure to an environment under high antibiotic selection pressure, whereas an index less than or equal to 0.2 indicates a low selective pressure environment [18, 19].

### 2.6. Statistical Data Analysis

Data from this study were saved into Excel 2019 software and analyzed using R Version 4.5.0 software. Qualitative variables are expressed as frequencies and percentages and presented in tables and figures. Quantitative variables are expressed as mean ± standard deviation and extreme values (min–max). Comparisons between groups were performed using the Mann–Whitney *U* test for quantitative variables. All analyses were considered statistically significant at *p*‐values less than 0.05.

## 3. Results

A total of 196 strains of *E. coli*, *E. cloacae*, and *K. pneumoniae* were included in the study, including 60 strains found in outpatients with asymptomatic carriage (carriage strains) and 136 clinical strains were isolated from patients with infections (clinical strains). All carriage strains were obtained from stool samples, whereas clinical strains were obtained from various biological samples, including urine (*n* = 53), blood (*n* = 65), and pus (*n* = 18) (Table 1).

**Table 1:** Distribution of *Escherichia coli*, *Klebsiella pneumoniae*, and *Enterobacter cloacae* in carriage and clinical isolates.

Sources	*E. coli*, *n* (%)	*K. pneumoniae*, *n* (%)	*E. cloacae*, *n* (%)	Total
Carriage strains				
Fecal swab	37 (61.67)	17 (28.33)	6 (10.00)	60
Clinical strains				
Urine	37 (69.81)	15 (28.30)	1 (1.89)	53
Blood	19 (29.23)	32 (49.23)	14 (21.54)	65
Pus	10 (55.56)	7 (38.89)	1 (5.56)	18
Total	103	71	22	196

### 3.1. Antibiotic Resistance Profiles

Resistance profiles were analyzed for 196 strains of *E. coli* (*n* = 103), *E. cloacae* (*n* = 22), and *K. pneumoniae* (*n* = 71) obtained from both asymptomatic outpatients and clinical infections. Resistance rates were analyzed and compared according to source (carriage vs. clinical strains) and species. The results of this study did not include natural resistance to aminopenicillins or first‐generation cephalosporins in *E. cloacae* or *K. pneumoniae*.

### 3.2. Resistance Profiles by Antibiotic Class

**Figure 2:**
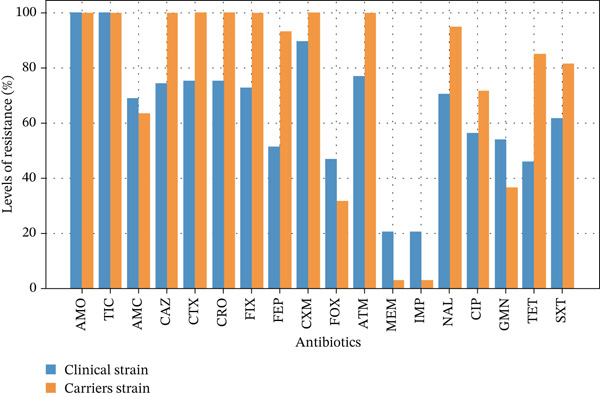
Levels of resistance to different antibiotics in carriage and clinical strains.

Analysis of resistance profiles revealed high resistance levels in both groups of strains for most antibiotic classes tested. Penicillins, cephalosporins, and monobactams presented particularly high levels of resistance, especially in carriage strains of *E. coli*. In contrast, resistance to cephamycins, carbapenems, and aminoglycosides was lower among carriage strains than among clinical strains. High resistance to quinolones, TETs, and sulfonamides was observed, particularly among carriage strains. These findings are shown in Figure 2.

Statistical comparison of resistance levels between carriage and clinical strains for each antibiotic class revealed the following: aminoglycosides (*p* = 0.04, 95% CI: −31.8 to −2.2), carbapenems (*p* < 0.001, 95% CI: −25.4 to −9.1), cephalosporins (*p* = 0.03, 95% CI: 3.0–11.7), quinolones (*p* < 0.001, 95% CI: 8.2–25.9), monobactams (*p* = 0.01, 95% CI: 6.7–25.6), penicillins (*p* = 1.00, 95% CI: 0.0–0.0), sulfonamides (*p* = 0.01, 95% CI: 7.2–32.7), and TETs (*p* < 0.001, 95% CI: 27.1–51.7). These results indicate that carriage strains showed significantly higher resistance to cephalosporins, quinolones, monobactams, sulfonamides, and TETs, whereas clinical strains showed significantly higher resistance to aminoglycosides and carbapenems. No difference was observed for penicillins (*p* = 1.00). Table 2 shows the differences in the resistance levels of strains to different classes of antibiotics from different sources. However, the differences were not statistically significant (*p* = 0.23).

**Table 2:** Comparison of antibiotic resistance patterns between carriage and clinical strains.

Antibiotic class	Carriage strains (%) mean ± SD	Clinical strains (%) mean ± SD	Difference (*Δ*)	95% CI	*p*
Aminoglycosides	36.67 ± 0.00	53.68 ± 0.00	−17.01	−31.8 to −2.2	0.04
Carbapenems	3.33 ± 0.00	20.59 ± 0.00	−17.25	−25.4 to −9.1	< 0.001
Cephalosporins	89.29 ± 32.76	69.34 ± 17.62	7.35	3.0–11.7	0.03
Quinolones	83.35 ± 9.51	63.25 ± 6.00	17.06	8.2–25.9	< 0.001
Monobactams	100.00 ± 0.00	77.20 ± 0.00	16.13	6.7–25.6	0.01
Penicillins	87.77 ± 17.30	89.70 ± 14.57	0.00	0.0–0.0	1.00
Sulfonamides	81.67 ± 0.00	61.76 ± 0.00	19.90	7.2–32.7	0.01
Tetracyclines	85.00 ± 0.00	45.60 ± 0.00	39.41	27.1–51.7	< 0.001

### 3.3. Resistance According to Bacterial Species

Carriage strains of *E. coli* were almost all resistant to cephalosporins, monobactams, quinolones, TETs, and sulfonamides, whereas the clinical strains presented lower resistance. However, AMC, cephamycin, and carbapenems were more frequently observed in clinical strains than in carriage strains. For *E. cloacae*, most antibiotics tested showed resistance among carriage strains, whereas resistance rates varied among clinical strains. However, carbapenems remained active in both strain groups. For *K. pneumoniae*, carriage strains were more resistant to antibiotics than clinical strains for all antibiotics tested, except for carbapenems, to which carriage strains were completely sensitive. The resistance levels of the bacterial strains are presented in Table 3.

**Table 3:** Antibiotic resistance profiles, ESBL production, and multidrug resistance distribution in carriage and clinical strains.

Parameters	Carriage strains			Clinical strains		
	*E. coli* *N* = 37 (61.67%)	*E. cloacae* *N* = 6 (10.00%)	*K. pneumoniae* *N* = 17 (28.33%)	*E. coli* *N* = 66 (48.53%)	*E. cloacae* *N* = 16 (11.76%)	*K. pneumoniae* *N* = 54 (39.71%)
Antibiotics tested (*n* = 196)						
AMO	37 (100.00)	RN	RN	66 (100.00)	RN	RN
TIC	37 (100.00)	RN	17 (100.00)	66 (100.00)	RN	54 (100.00)
AMC	14 (37.84)	6 (100.00)	RN	24 (36.36)	16 (100.00)	RN
CXM	37 (100.00)	6 (100.00)	17 (100.00)	63 (95.45)	16 (100.00)	43 (79.63)
FOX	7 (18.92)	RN	7 (41.18)	25 (37.88)	RN	23 (42.59)
CAZ	37 (100.00)	6 (100.00)	17 (100.00)	59 (89.39)	11 (68.75)	31 (57.41)
CTX	37 (100.00)	6 (100.00)	17 (100.00)	59 (89.39)	12 (75.00)	31 (57.41)
CRO	37 (100.00)	6 (100.00)	17 (100.00)	59 (89.39)	12 (75.00)	31 (57.41)
FIX	37 (100.00)	6 (100.00)	17 (100.00)	59 (89.39)	9 (56.25)	31 (57.41)
FEP	34 (91.89)	6 (100.00)	17 (100.00)	41 (62.12)	9 (56.25)	20 (37.04)
ATM	34 (91.89)	6 (100.00)	16 (94.12)	60 (90.91)	10 (62.50)	35 (64.81)
IMP	2 (5.41)	0 (0.00)	0 (0.00)	20 (30.30)	0 (0.00)	8 (14.81)
MEM	2 (5.41)	0 (0.00)	0 (0.00)	20 (30.30)	0 (0.00)	8 (14.81)
GMN	12 (32.43)	2 (33.33)	8 (47.06)	43 (65.15)	5 (31.25)	25 (46.30)
NAL	35 (94.59)	6 (100.00)	16 (94.12)	46 (69.70)	12 (75.00)	37 (68.51)
CIP	27 (72.97)	6 (100.00)	10 (58.82)	43 (65.15)	9 (56.25)	24 (44.44)
TET	31 (83.78)	5 (83.33)	15 (88.24)	29 (43.94)	9 (56.25)	24 (44.44)
SXT	30 (81.08)	5 (83.33)	14 (82.35)	51 (77.27)	6 (37.50)	27 (50.00)
ESBL production (*n* = 196)						
ESBL	26 (70.27)	1 (16.67)	7 (41.18)	59 (89.39)	9 (56.25)	31 (57.41)
Profile of ESBL strains (*n* = 133)						
NAL	26 (100.00)	1 (100.00)	7 (100.00)	42 (71.19)	7 (77.78)	21 (67.74)
CIP	21 (80.77)	1 (100.00)	3 (42.86)	41 (69.49)	5 (55.56)	1 (35.48)
GMN	10 (38.46)	0 (0.00)	5 (71.43)	42 (71.19)	2 (22.22)	15 (48.39)
TET	22 (84.62)	1 (100.00)	7 (100.00)	25 (42.37)	5 (55.56)	12 (38.71)
SXT	22 (84.62)	1 (100.00)	7 (100.00)	46 (77.97)	2 (22.22)	13 (41.94)
Multidrug resistance profile (*n* = 196)						
Non‐MDR	0 (0.00)	2 (33.33)	0 (0.00)	2 (3.03)	0 (0.00)	8 (14.81)
MDR	35 (94.60)	4 (66.66)	17 (100.00)	53 (80.30)	15 (93.75)	46 (85.19)
XDR	2 (5.40)	0 (0.00)	0 (0.00)	9 (13.64)	1 (6.25)	0 (0.00)
PDR	0 (0.00)	0 (0.00)	0 (0.00)	2 (3.03)	0 (0.00)	0 (0.00)

Abbreviations: AMC, amoxicillin–clavulanic acid; AMO, amoxicillin; ATM, aztreonam; CAZ, ceftazidime; CIP, ciprofloxacin; CTX, cefotaxime; CXM, cefuroxime; ESBL, extended‐spectrum beta‐lactamase; FEP, cefepime; FIX, cefixime; FOX, cefoxitin; GMN, gentamicin; IMP, imipenem; MDR, multidrug‐resistant; MEM, meropenem; NAL, nalidixic acid; PDR, pandrug‐resistant; RN, resistant naturally; SXT, cotrimoxazole; TET, tetracycline; TIC, ticarcillin; XDR, extensively drug‐resistant.

### 3.4. ESBL Production

ESBL profile revealed that 133 strains (67.86%) produced these enzymes. Among the carriage strains, 56.67% produced ESBL, whereas 72.79% of the clinical strains produced ESBL, a statistically significant difference (*p* = 0.02). Among the species, the majority of the producing strains were *E. coli*, followed by *K. pneumoniae* and *E. cloacae* (Table 3).

In addition, these strains presented relatively high resistance rates to other families of antibiotics, excluding beta‐lactams, regardless of their source.

### 3.5. Distribution of Multidrug Resistance Profiles and MAR Index

Resistance phenotype analysis of the 196 strains (carriage and clinical strains combined) revealed that 86.73% (170/196) were resistant to at least three classes of antibiotics tested (MDR). Among the remaining strains, 6.12% (12/196) were resistant to most classes of antibiotics tested except two or less (XDR), and 1.02% (2/196) were resistant to all classes of antibiotics tested (PDR).

Carriage strains had a greater proportion of MDR strains (93.33%, 56/60) than clinical strains (87.50%, 119/136) but not statistically significant (*p* = 0.225). Likewise, no statistical significance was found in the XDR (*p* = 0.46) and PDR (*p* = 0.346) profiles despite more clinical strains exhibiting such trait. Notably, XDR and PDR profiles were observed exclusively in *E. coli* clinical strains (Table 3).

**Figure 3:**
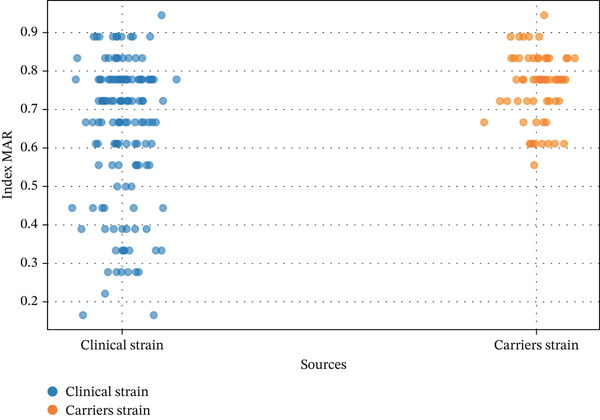
Distribution of the multiple antibiotic resistance (MAR) index in strains based on source.

MAR index analysis revealed variable results. The MAR index for carriage strains ranged from 0.57 to 0.94 (mean 0.76 ± 0.09), whereas for clinical strains, it ranged from 0.17 to 0.94 (mean 0.65 ± 0.18) (Figure 3). Analysis of the indices between the clinical and carriage strains revealed a significant difference according to the Mann–Whitney *U* test (*p* = 0.012).

## 4. Discussion

In this study, main species studied were *E. coli*, *K. pneumoniae*, and *E. cloacae*. These bacterial species are frequently associated with bacterial infections and are the most commonly found in carriage studies [20].

The majority of strains in both groups (clinical and carriage strains) in our study were *E. coli*, which is similar to previous reports in the literature showing that this bacterial species is both a commensal of the human digestive tract and an opportunistic pathogen. As such, it plays a crucial role in the gut microbiota and is frequently associated with digestive and urinary tract infections and sepsis [13, 21].

Moreover, the lower representation of *K. pneumoniae* and *E. cloacae* in the bacterial populations investigated could be explained by their lower density in the digestive tract compared with that of *E. coli* [23]. *K. pneumoniae* and *E. cloacae* have also been found more frequently among clinical strains than among carriage strains, particularly those isolated from urine and blood samples, reflecting their opportunistic nature and role in hospital‐acquired infections. These findings reinforce observations reported in other studies conducted in sub‐Saharan Africa, where *K. pneumoniae* and *E. cloacae* are more often associated with nosocomial infections [13, 24, 25].

Phenotypically, the results of this study revealed different resistance profiles between the carriage and clinical strains. Carriage strains were more resistant to cephalosporins, fluoroquinolones, monobactams, TETs, and sulfonamides, with a higher MAR index (mean of 0.76 ± 0.09).

This observation may suggest increased antibiotic exposure and possible selective pressure in community settings, probably associated with the lack of regulation on access to antibiotics, which encourages self‐medication, inappropriate usage in intensive animal production, and indirect exposure through food via antibiotic residues in meat and vegetables purchased from markets. These results corroborate those of several studies in which high levels of resistance have been associated with excessive antibiotic use in the community [13, 26–29]. In contrast, clinical strains presented higher rates of resistance to aminoglycosides (53.68% vs. 36.67%) and especially to carbapenems (20.59% vs. 3.33% in carriage strains), with an average MAR index of 0.65 ± 0.18. These results may reflect differences in antibiotic usage practices in hospital settings, where these molecules are used more frequently to treat serious infections. The alarming levels of carbapenem resistance observed in clinical strains suggest the possible emergence of carbapenemase‐producing strains, which is consistent with the phenotypic trends described in certain studies conducted on clinical strains in Côte d′Ivoire. For example, in a study published in 2024, Yapi et al. [30] reported that 28.49% of 171 Enterobacteriaceae strains isolated from confirmed human infections in Côte d′Ivoire were resistant to carbapenems.

The higher resistance observed in carriage strains for certain antibiotic classes (cephalosporins, fluoroquinolones, and monobactams) may be explained by the widespread circulation of mobile genetic elements, particularly IncF and IncI plasmids, which carry ESBL genes (notably *bla*_CTX-M-15_) along with resistance determinants for quinolones (*qnr* genes) and aminoglycosides. Similar findings have been reported in long‐term care facilities in Europe, where colonization pressure and antibiotic exposure drive the persistence of ESBL‐producing Enterobacterales [31].

On the other hand, in a newly established hospital setting, carbapenemase‐producing Gram‐negative bacteria have been documented as emerging threats, highlighting the importance of early surveillance [32]. Furthermore, the presence of carbapenem‐resistant strains in our clinical isolates (20.59%) raises concern about possible carbapenemase producers such as NDM or OXA‐48, which have been reported in Africa. In this context, fosfomycin has been described as a therapeutic option for infections caused by NDM‐5–producing *E. coli* [33], though it was not tested in our study.

The analysis of the resistance profiles obtained in our study, when subjected to the criteria of Magiorakos et al., revealed a high prevalence of MDR strains among the examined strains. This observation suggests a widespread distribution of Enterobacteriaceae resistant to at least three major classes of antibiotics in the community (93.33%) and hospitals (87.50%). Only a minority of strains (6.12%) were sensitive to most classes of antibiotics tested. Our results are similar to those of a study conducted in Ethiopia, which revealed a high overall rate (92.10%) of MDR Enterobacteriaceae strains among 164 Enterobacteriaceae strains [34]. For the species investigated, our study revealed a high proportion of MDR strains among the three Enterobacteriaceae species studied. This profile was observed in 85.44%, 86.36%, and 88.73% of *E. coli*, *E. cloacae*, and *K. pneumoniae* strains, respectively. Our findings highlight the spread of resistance regardless of bacterial species and suggest a role for these strains in the spread of resistance in community and hospital settings [35]. In addition, our study revealed strains with XDR and PDR profiles in lower proportions (6.12% and 1.02%, respectively). Although rare, these strains pose a major public health risk not only because of the limited number of effective antibiotics but also because of the risks of therapeutic impasses and increased mortality associated with them. Recent studies conducted in the Middle East and other regions, which focused on infections caused by XDR Enterobacteriaceae strains, reported mortality rates ranging from 50% to 84% and prolonged hospital stays with an average duration of 27 days [36, 37]. In Africa, although causality studies are less common, some studies have indicated that the presence of XDR strains complicates treatment regimens and increases mortality [38].

Although less frequent, the XDR profiles were found only in *E. cloacae* (13.64%) and *E. coli* (10.68%) but none from *K. pneumoniae* in our study. The presence of XDR profiles in *E. cloacae* could be due to its intrinsic ability to acquire plasmids that confer multidrug resistance [39, 40]. In *E. coli*, these profiles may reflect the selection of resistant clones actively circulating in the community, particularly in urban areas with high antibiotic pressure. PDR strains were observed only in *E. coli* (1.94%). Although this proportion is low, it could play a role in the harboring and transfer of resistance genes. Studies by Pan et al. [41] in 2023 and Kaur et al. [42] in 2024 reported the presence of ST38 and ST167 clones of PDR *E. coli* carrying genes conferring resistance to all current classes of antibiotics, including polymyxins, owing to the presence of several genes encoding efflux pumps. For both sources, MDR strains were largely dominant, with higher proportions from clinical strains than carriage strains. This finding highlights the silent community circulation of multidrug resistance, which may be fueled by the risk factors such as large household size living in a single room, presence of children under 15 years old, previous hospitalization, antibiotic use, and chronic medical diseases, associated with intestinal carriage in a previous study [43]. Likewise, XDR and PDR profiles were more common in clinical strains than in carriage strains. This difference could be linked to more intense selection pressure in hospital settings. These strains are often associated with prolonged hospital stays and invasive procedures [36].

Faced with the high prevalence of MDR and emerging XDR/PDR strains observed in this study, new therapeutic options are under development or have recently been approved. Recent in vitro studies have demonstrated promising activity of novel *β*‐lactam/*β*‐lactamase inhibitor combinations against MDR Enterobacteriaceae. Specifically, FEP–enmetazobactam, FEP–taniborbactam, and FEP–zidebactam have shown potent activity against ESBL producers and various carbapenemase classes [44]. Additionally, cefiderocol (a siderophore cephalosporin) and the combination ATM–avibactam have demonstrated effectiveness against metallo‐*β*‐lactamase–producing *E. coli*, including NDM producers [45]. These molecules could represent a promising hope in the fight against emerging MDR strains, although their availability in low‐resource settings such as Côte d′Ivoire remains limited because of high costs and regulatory constraints.

## 5. Limitations

This study has several limitations. First, fecal samples from outpatients were self‐administered rectal swabs, so improper sampling technique cannot be ruled out. Second, polymyxins (colistin) were not included in antibiotic susceptibility testing, limiting our ability to detect resistance to this last‐resort antibiotic class. Another limitation of this study is the use of phenotypic methods for ESBL detection based on synergy testing. Although widely used in routine microbiology laboratories, the sensitivity and specificity of this approach may vary, particularly in the presence of other resistance mechanisms such as AmpC *β*‐lactamases or carbapenemases. In addition, phenotypic testing does not allow identification of specific resistance genes, which may lead to underestimation or misclassification of ESBL‐producing isolates.

Third, the study was restricted to two university hospitals in Abidjan, which may limit generalizability to other regions of Côte d′Ivoire. Fourth, sample sizes for certain species (*E. cloacae*) were small, potentially reducing statistical power. Finally, molecular characterization of resistance genes was not performed, precluding identification of specific genetic determinants.

## 6. Conclusion

The findings of this study highlight the significant circulation of MDR strains of Enterobacteriaceae in community and hospital settings and the role of both asymptomatic carriage and clinical infections in the spread of resistance. The differences observed between the antibiotic resistance profiles of strains carried by asymptomatic outpatients and clinical strains isolated from bacterial infections in hospitalized patients reflect distinct selection pressure. The high representation of MDR strains in both carriage and clinical strains, as well as the presence of XDR and PDR strains among clinical strains, although at low frequencies, highlights the risk of the emergence of difficult‐to‐treat infections, increasing the importance of integrated and targeted surveillance strategies. These findings call for a comprehensive approach combining microbiological surveillance, control of antibiotic use, and prevention of human‐to‐human transmission to limit the spread of MDR Enterobacteriaceae strains in urban areas.

## Author Contributions

All authors contributed to the study conception and design. Material preparation, data collection, and analysis were performed by K.K.A.A., K.K.S., Y.I.A., O.D.N.C., B.A., and A.F. The first draft of the manuscript was written by K.K.A.A. and K.K.S.

## Funding

No funding was received for this manuscript.

## Disclosure

All authors commented on previous versions of the manuscript. All authors read and approved the final manuscript.

## Conflicts of Interest

The authors declare no conflicts of interest.

## Data Availability

The data that support the findings of this study are available from the corresponding author upon reasonable request.
